# Investigation into a Superspreading Event of the German 2020–2021 Avian Influenza Epidemic

**DOI:** 10.3390/pathogens11030309

**Published:** 2022-03-02

**Authors:** Nicolai Denzin, Marlies Bölling, Anne Pohlmann, Jacqueline King, Anja Globig, Franz Josef Conraths

**Affiliations:** 1 Friedrich-Loeffler-Institut, 17493 Greifswald, Germany; anne.pohlmann@fli.de (A.P.); jacqueline.king@fli.de (J.K.); anja.globig@fli.de (A.G.); franz.conraths@fli.de (F.J.C.); 2 Office for Consumer Protection and Veterinary Affairs, District of Paderborn, 33102 Paderborn, Germany; boellingm@kreis-paderborn.de

**Keywords:** highly pathogenic avian influenza, H5N8, epidemiology, transmission, poultry, travel trade

## Abstract

Between November 2020 and May 2021, Germany faced the largest highly pathogenic avian influenza (HPAI) epidemic recorded so far with 245 outbreaks in poultry and captive birds and more than 1000 diagnosed cases in wild birds. In March 2021, an HPAI outbreak of subtype H5N8 was confirmed in a holding rearing laying hens for sales. Disease introduction probably occurred via indirect contact with infected wild birds. Since the index farm sold chicken to customers including many smallholders, partly in travel trade, the primary outbreak triggered 105 known secondary outbreaks in five German federal states. An outbreak investigation was carried out with links between the involved farms retrieved from the German Animal Disease Notification System used for network analysis. In some cases, links were confirmed through sequence-based molecular analysis. Special emphasis was put on the estimation of the flock incubation period as a prerequisite of sound contact tracing. The unique circumstances of an outbreak farm with frequent direct trade contacts prior to disease suspicion enabled an assessment of the flock incubation period based on the consequences of contacts, further supported by molecular analysis and modeling of disease spread. In this case, the flock incubation period was at least 14 days.

## 1. Introduction

Since 2006, when highly pathogenic avian influenza (HPAI) H5 viruses from the goose/Guangdong (gs/GD) lineage first caused deaths in wild birds and poultry in Germany, numerous epidemics associated with H5 HPAI virus of the same lineage have been recorded in different years. In autumn 2020, the introduction of HPAI viruses (H5N8 and H5N5) subsequently triggered the largest HPAI epidemic ever recorded in Germany and had a severe impact on wild bird populations and the poultry production sector [1]. For the first time, the epidemic did not completely fade out during the summer months in Europe, with ongoing detections in wild birds throughout 2021 [2]. Although the H5 HPAI viruses of 2016/2017 and 2020 (to date) belong to the same clade of 2.3.4.4b, they are not directly phylogenetically linked and probably display separate introductions to Germany ([3,4]).

In Germany, the reporting of suspicion and confirmation of notifiable animal diseases to a central database, the German animal disease notification system (Tierseuchen-nachrichtensystem, TSN, [5]), is mandatory. The database is hosted by the Federal Research Institute for Animal Health (Friedrich-Loeffler-Institut, FLI, [6]).

More than 1000 cases in wild birds and a total of 245 outbreaks in poultry and captive birds were recorded in TSN between November 2020 and May 2021. Within a short period between late March and early April, over 100 new outbreaks occurred in poultry, all due to the secondary spread of the virus. Originating from a poultry farm in the district of Paderborn (North Rhine-Westphalia), H5N8 HPAIV spread mainly to small holdings in Baden-Württemberg and Thuringia through the distribution of live poultry via trade routes [7].

According to the current legislation, outbreak investigation in case of notifiable animal diseases, such as HPAI, has to be carried out by the veterinary authority of the respective district. Support by the outbreak investigation unit of the FLI may be sought.

Here, the outbreak of HPAI in the district of Paderborn was investigated by the respective veterinary authority in conjunction with the FLI involving classical outbreak investigation on the farm with interviewing and assessment of the documentation, analysis of the TSN database, molecular analysis, and a modeling approach of the disease spread on the farm.

The presented study unravels contact patterns in a complex epidemiological scenario of an outbreak on a farm raising laying hens for travel trade with an unprecedented number of secondary outbreaks. Special emphasis was put on the determination of the herd incubation period, which is of utmost importance for contact tracing in disease control.

## 2. Results

### 2.1. Outbreak Investigation and Test Results

**Figure 1:**
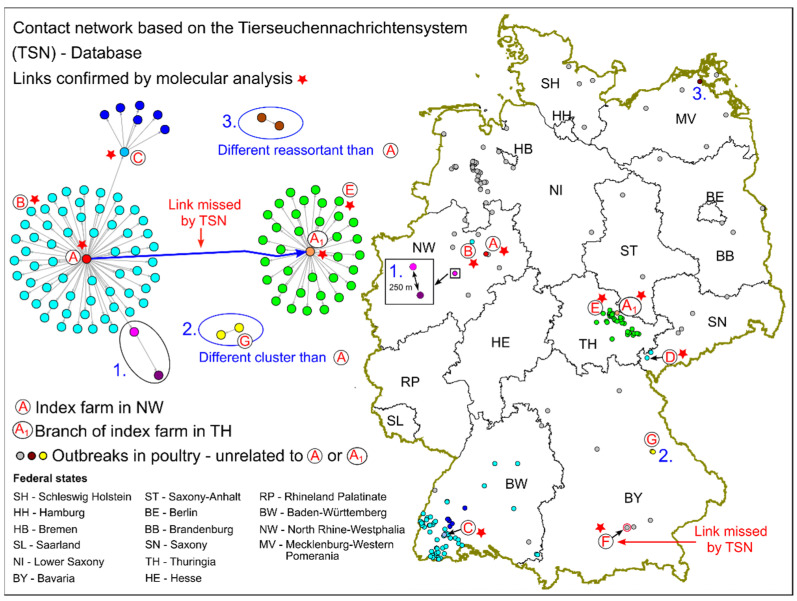
Outbreaks of highly pathogenic avian influenza in poultry in Germany between 1 March and 12 July 2021—location and epidemiological links.

The primary outbreak farm (farm A, Figure 1) is located in the German federal state of North Rhine-Westphalia (NW) in an area of high poultry density. The farm is a family business specialized in the rearing and selling of laying hens. Altogether, about 30,000 hens were kept in three compartments of a closed stable in age groups of 2, 7, and 19 weeks at the time of outbreak detection. An outbreak of HPAI was clinically suspected on 20 March 2021 (Figure 4, day zero) in the oldest age group (19 weeks) with 15 hens of about 10,500 succumbing during 24 h. A penside rapid test (FASTest^®^ AIV Ag, Megacor Diagnostik GmbH, Hörbranz, Austria) for influenza A, carried out by a private practitioner on suspicious animals, had a positive result. The veterinary authority of the district was notified the same day. It declared a suspected outbreak of HPAI, ordered a stand-still for the farm and established restriction zones with clinical examination or sampling of relevant holdings, and took samples in each epidemiological unit of the suspect farm (combined cloacal-pharyngeal swabs of all age groups, *n* = 97; blood samples from the group with 19-week-old chickens, *n* = 20). Only in samples of the 19-week-old hens was influenza A, subtype H5, confirmed by the regional veterinary investigation center the following day (Figure 4, day 1). Out of 35 swabs (from 35 diseased hens), 30 tested positive by RT-qPCR. This corresponds to a prevalence of 85.7% (CI_95_: 69.7, 95.2 according to Clopper-Pearson, in [8]). Of the 20 blood samples taken from clinically healthy birds of the same age group (19 weeks) only one tested positive for influenza H5—specific antibodies. An outbreak of HPAI, subtype H5N8, was confirmed by the National Reference Laboratory for Avian Influenza at the Friedrich-Loeffler-Institut, Federal Research Institute for Animal Health, Greifswald-Insel Riems, Germany, on day 2 (Figure 4). All chicken kept on the premises were culled the same day.

### 2.2. Epidemiology

#### 2.2.1. Epidemiological Investigation

The biosecurity and biosafety measures in place on the affected farm (farm A) were found to be sound. No recent contacts with other infected premises could be identified as a source of infection. Indirect contact with infected wild birds was considered the most likely route of infection, i.e., possible introduction of infectious material via the staff lock, or ventilation system. The identification of holdings to which the disease could have spread from farm A (tracing-on) was facilitated by the fact that the trading activity of the farm had commenced only 14 days before the disease outbreak was suspected (Figure 4), thus limiting the maximal time span to be considered.

Within this period, a total of 307 farms and small holders were provided with 2743 chickens originating from farm A. The animals were partly delivered directly to the customer as one individual batch, especially when larger numbers of birds were ordered. Alternatively, the chickens left the outbreak farm A as a batch and were then channeled through farm A_1_, a branch farm of outbreak farm A in Thuringia (TH, same owner), and further distributed from the latter, or they were sold to small holders via sales tours (travel trade). These direct contacts, i.e., the movement or selling of poultry from farms A and A_1_ to other poultry holdings led to 98 direct secondary outbreaks.

All traced contacts, including confirmed outbreaks due to further spread from the initial direct secondary outbreaks of farms A and A_1_, amounted to 314 farms in six German federal states and ultimately led to 105 outbreaks in five federal states. One of the aforementioned further outbreaks was most likely due to the close vicinity of the affected farm to a direct secondary outbreak of farm A. Further trade of chickens from a farm in Baden-Württemberg (BW), which had received chickens from farm A, led to six additional secondary outbreaks. Details of the traced contacts and outbreaks are provided in Figure 1 and Table 1. No chickens were sold abroad.

Most contacts were identified for the federal states of TH and BW, where the proportion of secondary outbreaks resulting from contacts was 57% (BW) and 19% (TH), respectively.

The earliest direct contact took place on day -14: On this day, a small holder (Figure 1, farm B) received 150 chickens from farm A. Farm B suffered an outbreak of HPAI that was suspected the same day the disease became evident on farm A (Figure 4, day zero). Based on the first documented losses on farm A excluding background losses of a few birds (Figure 4) on day -2 and an assumed individual incubation period of 3–5 days [9], it was initially thought that disease introduction on farm A had occurred around day -7 or only slightly earlier. Therefore, special emphasis was placed on the substantiation of causation concerning secondary outbreaks attributed to earlier contacts of outbreak farm A, particularly the outbreak on farm B.

**Figure 2:**
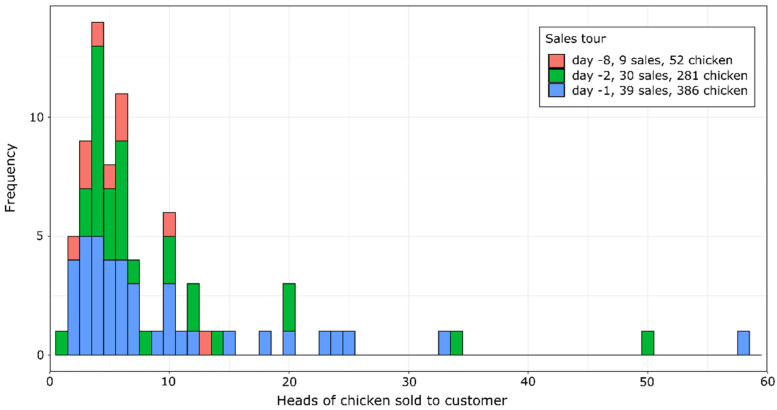
Numbers of chickens sold to customers on three sales tours to BW (in total 78 sales and 719 chickens).

Figure 2 illustrates the distribution of the number of chickens sold to customers on the three distinct sales tours to BW (Figure 1). The majority of customers were small holders purchasing less than 10 chickens per sale.

#### 2.2.2. Network Analysis

Information retrieved from the German Animal Disease Notification System (Tier-seuchennachrichtensystem, TSN, [5]), revealed two major clusters of secondary outbreaks, one originating from contacts of the primary outbreak farm A, the other from the branch farm A_1_ in TH (Figure 1). The first cluster continued to spread through secondary trade contacts (farm C) and, in one case, probably through the vicinity to another outbreak (“1.”). The link between farm A and A_1_ was originally missing in the database, as it only became evident after a detailed analysis of the course of disease and trade connections between the two farms. The link of an outbreak in Bavaria (BY, Figure 1, “F”) to farm A or A_1_ was also only later revealed via molecular analysis. In a follow-up investigation, the veterinary authority of the respective district (Erding) in which farm F is situated disclosed that laying hens purchased by the poultry holder prior to the outbreak had originated from a garage sale in Erding which, according to unconfirmed information, represented the end of a trade cascade that probably started in Northern Germany.

**Figure 3:**
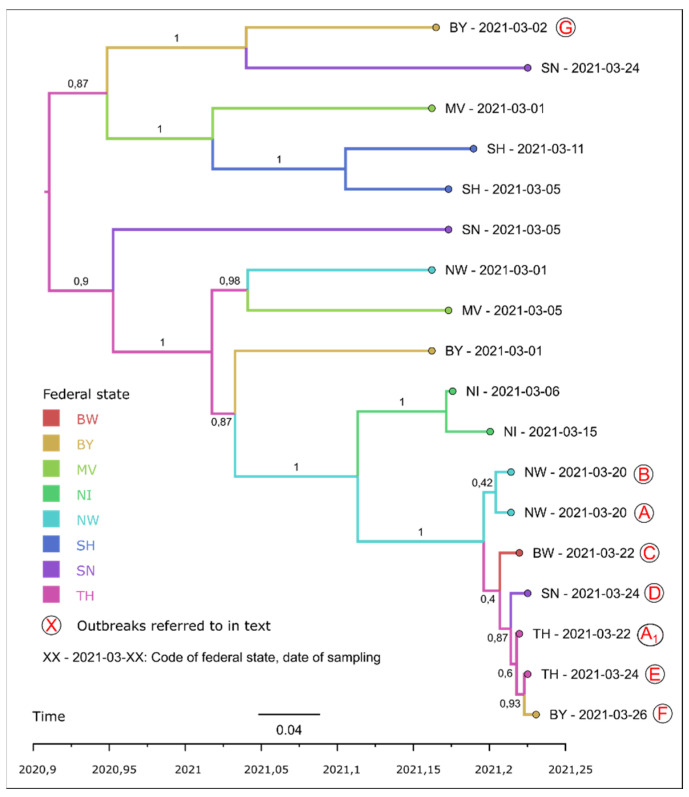
Time-scaled tree of the genomic variation of samples of outbreaks of HPAI, subtype H5N8 in chickens in March 2021.

Network analysis further revealed two smaller clusters, comprising of only two outbreaks each, that were obviously unrelated to farms A and A_1_. The latter was confirmed by molecular analysis (see below). Trade contacts to NW were unlikely since the cluster in BY comprised a large parent flock for broiler production (Figure 1 and Figure 3, farm G) and a mixed geese and duck backyard holding (secondary outbreak of farm G). The other small cluster in Mecklenburg-Western Pomerania (MV) represented two small mixed holdings (chickens, ducks, and geese; geese and pigeons), which were kept in a single epidemiological unit, but with two different registration numbers.

#### 2.2.3. Sequence Based Molecular Analysis

**Figure 4:**
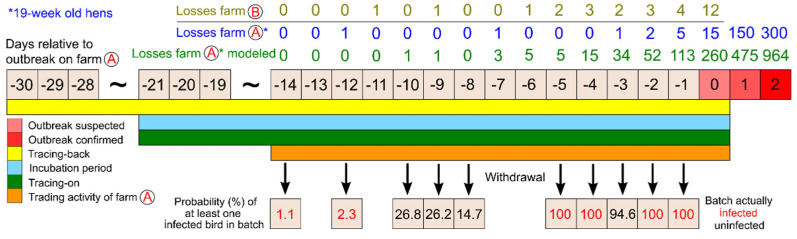
Timeline of events related to the outbreak on farm A, modeled losses and probability of trade batch infection; losses on farm B.

Molecular analysis via phylogenetic maximum likelihood (ML) and subsequent time-scaled maximum clade credibility (MCC) analysis of whole genome sequences was carried out for a selection of 18 genomes from outbreaks of HPAI, subtype H5N8, collected from chicken holdings in March 2021 in several federal states in Germany. Seven virus genomes of this selection form a cluster indicating strong phylogenetic relatedness (Figure 3, marked A–F). This is also reflected by the high similarity at nucleic acid level with 99.97–99.91% identity or 3–13 positional differences across the entire coding sequences. These seven clustered genomes were clearly distinct from other virus genomes obtained from other chicken farms in Germany in the same month (28–107 positional differences over entire coding sequences), and virus genomes of index farm A (NW) and branch farm A_1_ (TH) were part of the cluster, highlighting their role in the superspreading event. As expected, virus genomes from farm A and its branch farm A_1_ were closely related. The analysis also revealed a close relation between virus genomes from farms A and farm B (both located in NW) (Figure 1 and Figure 3), which had received chickens from farm A already on day -14 (Figure 4). Moreover, the MCC analysis and the subsequently calculated phylogeographical diffusion models showed that virus genomes from farm C (Baden-Württemberg, BW) were linked to farm A, whereas the virus genomes from farms D (Saxony, SN), E (Thuringia, TH), and F (Bavaria, BY) were connected to the branch farm A_1_ in Thuringia. The spatio-temporal order of the outbreaks with farm A connected to farm B (NW), C (BW) and branch farm A_1_ (TH), and branch farm A_1_ subsequently linked to E (TH), D (SN), and F (BY) was confirmed by the phylogeographical diffusion models.

A link between the index case on farm A or the branch farm A_1_, respectively, with farm G (BY), could be excluded by molecular analysis. The virus genomes obtained from farm G belonged to a different cluster than those retrieved from farms A and A_1._ Similarly, an isolate from Mecklenburg-Western Pomerania (Figure 1, contact cluster “3.”), which even constituted a reassorted virus (data not shown) was different from the virus genomes recovered from farms A and A_1_ and all other studied farms.

### 2.3. Modeling of Within-Farm Spread

To analyze the within-farm spread of HPAI in the outbreak compartment of index farm A with 19-week-old hens, a model was implemented in R [10]. We assumed that only one chicken was infected on day -14 (Table 2). Although conservative parameter estimates for the latency period, infectious period, and secondary attack rate were used in the model, the number of infected chickens (Table 2) and poultry that succumbed to HPAI (Figure 4) increased rapidly towards day zero, i.e., the day on which the outbreak was suspected on farm A.

The modeled losses on farm A clearly exceed the observed losses from day -7 onward (Figure 4). Figure 4 also illustrates the observed losses on farm B, which had received 150 laying hens from farm A on day -14. Excess losses seem to have occurred on farm B a few days earlier than on farm A.

### 2.4. Probability of Infection

Batches of chickens left the index farm on different days from day -14 until the outbreak was suspected, i.e., day zero. The probability that at least one bird of the chicken batch removed from farm A was infected, was very low at the beginning of the time span, however resulting in secondary outbreaks in holdings that had received chickens from the respective batch (Table 2, Figure 4). Batches removed from day -10 to -8 had moderate probabilities of infection but did not lead to (known) further outbreaks. Close to day zero, the probabilities of infection reached 100%, which matched with traced secondary outbreaks originating from the respective batches.

Incubation period, tracing-back and tracing-on periods indicated in Figure 4 were worst-case assumptions for the practical disease control concerning the outbreak on farm A.

## 3. Discussion

In outbreak investigations, the determination of the most likely time of disease introduction into the outbreak farm is of utmost importance. The latter defines the relevant time span of tracing-back (where did the disease come from) and tracing-on (where did it go to) and is thus crucial for successful disease control. Usually, the time of disease introduction is deduced from the onset of clinical symptoms or positive test results and the incubation period. The applied incubation period is then often an individual incubation period, i.e., the time elapsing from infection to clinical (or laboratory) evidence in individual specimens similar to observations made in experiments. It is widely accepted that the so-called herd incubation period is different, i.e., longer, than the individual incubation period. Especially in larger herds, symptoms, or losses of the initially infected animals, particularly if they were only mild, may be missed or misinterpreted as background losses due to unknown reasons or non-regulated animal diseases. When larger cohorts of infected animals reach the stage of clinical disease or die after initial unnoticed spread, the dramatic course of disease and a massive increase in the rate of losses may lead to an underestimation of the time that has elapsed since the disease was introduced. This is particularly true if only the individual incubation period determined in experimental infections is applied to assess the most likely period of disease introduction. Underestimating the herd incubation period and assuming a period of introduction that starts after the true time of introduction will jeopardize the success of practical disease control, since events of potential spread may be missed in the epidemiological outbreak analysis. Moreover, the increase of scientific knowledge concerning potential routes of disease introduction is curtailed.

The presented outbreak investigation offered the unique opportunity to gain insight into possible herd incubation periods of H5N8 HPAI. Based on the highly dynamic development of disease in the affected flock and the losses and considering the (individual) incubation period of three to five days as indicated by the Food and Agriculture Organization (FAO, [9]), a disease introduction around day -5 (Figure 4) was first suspected. Yet, a smallholder (farm B, Figure 1) that had received 150 chickens from outbreak farm A on day -14, the first day of trade, had suffered an outbreak of HPAI. Farm B had housed the chickens indoors in a closed mobile stable that was stocked for the first time with no other source of birds than farm A. We cannot completely rule out that farms A and B may have been infected by chance almost simultaneously and independently, e.g., through indirect contact with infected wild birds. This is unlikely, however, since there were no other outbreaks near farm B at this time and there were hardly any reports on HPAI in wild birds in the region where farm B is situated. Thus, it seems more likely that the outbreaks were directly linked, supported by the close genetic relationship of virus genomes isolated from the two farms. The dynamics of the documented losses on farms A and B were similar, but unusual losses were apparently detected a few days earlier on farm B than on farm A. This can potentially be explained by the fact that small holdings are sometimes easier to monitor for disease and losses.

As shown in Table 2 and Figure 4, the probability that the batch of chickens delivered from farm A to farm B on day -14 was infected was low, but the risk was not negligible. The veterinary authority of the district where farm B is situated reported that the disease developed slowly in the holding. A private practitioner had been consulted on day -5 and treated the flock against respiratory disease. Early infection of farm A is also supported by the tracing of two secondary outbreaks in Saxony (SN), which received birds from a batch that had left farm A on day -12. Since these outbreaks are geographically closely related to many secondary outbreaks derived from the branch farm A_1_ in Thuringia (Figure 1), it might be assumed that the outbreaks in SN were also related to branch farm A_1_, though according to the records farm A_1_ had received a batch of chickens from farm A for the first time on day -5 in the relevant period. Yet, investigations of the veterinary authority of the respective district in SN seem to confirm that the chickens had originated from a batch directly distributed by farm A and that these birds had left the farm on day -12. The probability of infection of this batch was also low, mirrored by the fact that only two of 29 holdings supplied with chickens from this batch subsequently experienced HPAI outbreaks. However, the fact that the virus from one of the outbreak farms (D) in SN was genetically more closely related to the virus from the branch farm A_1_ than from farm A may contradict the scenario outlined above. Maybe the chickens destined for SN were actually channeled through branch farm A_1._ There was at least one unconfirmed statement in the interviews of farm personnel indicating that some chickens from index farm A were already relocated to branch farm A_1_ on day -12.

The probabilities of batch infection calculated for the days prior to disease confirmation on farm A (Table 2, Figure 4) support the findings of the epidemiological outbreak investigations in respect to the batches. All batches with a calculated probability of 100% of infection with HPAI led to secondary outbreaks. A batch that originated from day -3 did not transmit disease, although the probability of infection was high but not 100%. This batch comprised only of a small number of chickens (25, Table 2).

The two federal states affected most as far as contacts with farm A and resulting secondary outbreaks are concerned were BW and TH. In the latter federal state, the proportion of secondary outbreaks relative to the experienced contacts was considerably lower than in BW, probably because the farms in this state mainly received chickens from a batch that left farm A on day -12, when the percentage of infected birds was still low on farm A (Table 2). On the contrary, many secondary outbreaks in BW were due to sales tours supplying many small holders with chickens from the batches of the days -2 and even -1, i.e., the day before the outbreak was suspected on index farm A.

The modeling of disease spread conducted for the 19-week-old laying hens on farm A served mainly as an orientation. The losses modeled for the days close to the confirmation date of the outbreak were considerably higher than the observed losses (Figure 4), although conservative assumptions were made for the model parameters. The latent period (time span from infection to infectiousness) was set to two days according to findings of [11] for laying hens infected with HPAI, subtype H5N8, in 2014. Data of [12] referring to the year 2016 imply, however, that the latent period may be as short as one day. The time span from infection to death was chosen as 4.5 days, i.e., the latent period plus an infectious period of 2.5 days based on a literature review of [13] concerning the situation in the year 2014. The investigations of [12] in the year 2016 suggest a markedly shorter infectious period. Concerning the secondary attack rate, the lowest value estimated by [13] for a laying hen farm (4.4) was used, although values up to 34.4 were found.

Thus, the use of parameter values less favorable for a slow spread of disease in the model would have further increased the discrepancies between the modeled and the observed losses, rendering an actual disease introduction into farm A considerably later than day -14 more likely. It also must be taken into account that HPAI isolates may be genetically related, even if farms A and B were independently infected via wild birds, if only limited virus passaging had occurred. Moreover, the attribution of outbreaks to the early batches that had left the index farm A might be spurious and the suspected secondary outbreaks could in fact have been caused by contact to infected wild birds. Yet, we consider this much less likely.

Although it is a matter of probabilities, the presented investigation seems to hint towards the possibility of a slow spread of HPAI in a chicken farm. The secondary attack rate might be lower than 4.4 on average and the herd incubation period (flock-level) as long as 14 days. These findings support the specification of the flock-level incubation period (14 days) of HPAI independent of the subtype as detailed in the Terrestrial Animal Health Code of the World Organization for Animal Health (OIE, [14]). It should be noted, however, that [13], who estimated the time span from disease introduction to detection of H5N8 for five laying hen farms, found flock-level incubation periods as short as 5.9 days on average, but with a maximum at 14.8 days for one particular farm.

The described outbreak occurred on a farm that sold young laying hens to poultry holders in many German federal states, also to small holders on sales tours. This business model implies the risk of a wide, distance-independent spread of HPAI if the farm of origin is infected, but disease not yet suspected. According to the relevant German legislation in force at the time of the outbreak [15], the veterinary authority may demand a clinical examination carried out by a veterinarian not earlier than four days prior to the sale of the chickens on sales tours. However, this is difficult to implement for practical reasons and the risk remains that chickens may not yet show symptoms when examined. In any case, an exact documentation of sales with precise contact data of all customers irrespective of the number of birds sold is mandatory to allow proper tracing of contacts and disease control in case of an outbreak.

The information collated in the course of the outbreak investigations reported here may be used to improve prevention measures for HPAI spread through travel trade of poultry. It also emphasizes the importance of determining the herd (or flock) incubation time for HPAI in poultry holdings as precisely as possible.

## 4. Materials and Methods

The federal states of Germany are the competent authorities for animal disease control according to European Union and German national legislation. They have delegated these duties to the district veterinary offices in the affected federal states. The epidemiological investigation on outbreak farm A and the tracing of contacts were thus carried out by an official veterinarian of the district veterinary office of Paderborn, partly assisted by an epidemiologist of the Friedrich-Loeffler-Institut, Federal Research Institute for Animal Health. The veterinary authorities of the districts where the contact holdings were situated investigated these contacts. Information on outbreaks (primary and secondary) was submitted to the German Disease Notification System (Tierseuchennachrichtensystem, TSN, [5]).

TSN was developed and is continuously advanced and maintained by the Federal Research Institute for Animal Health [6]. It comprises an online component mainly for disease notification and software for disease control management. The competent local veterinary authorities are obliged to notify animal diseases via this system if they are notifiable or reportable according to the relevant European community or national legislation. The database of the notification system may be queried, and data may be downloaded by entitled and registered users.

Network analysis and visualization was carried out employing the software R [10] with the package igraph [16]. Outbreak data (HPAI in poultry) from the time interval 1 March to 12 July 2021 were downloaded from the TSN database as a csv-file and read into R. Among the information that has to be provided with the notification is a statement on the epidemiological status of the affected holding, i.e., primary (index) versus secondary outbreak, and in the latter case, the registration number of the traced source of infection. The registration number of the outbreaks and of the identified source of infection were used to analyze the data with igraph [16].

The penside rapid test (FASTest^®^ AIV Ag, Megacor Diagnostik GmbH, Hörbranz, Austria) for influenza A was carried out according to the manufacturer’s recommendations [17]. Tracheal swabs were immersed in 1 mL of buffer diluent, the eluate was mixed and 280 µL was transferred to a test tube. A dipstick was added to the test tube and results were read after 20 min. Positive samples containing avian influenza virus type A antigens (subtypes H1–H15) react in the conjugate pad area of the dipstick with mobile monoclonal anti-AIV antibodies (anti-AIV mAbs), which are bound to colloidal gold particles. Migrating (“lateral flow”) along the nitrocellulose membrane, these specific antigen-antibody complexes are bound by fixed anti-AIV mAbs producing a pink-purple test line.

Confirmatory Polymerase Chain Reaction (PCR) testing of samples was conducted by matrix gene-based real-time reverse-transcription quantitative PCR (RT-qPCR) to screen for influenza A positive samples from the holding [18]. Subsequently, the latter samples were tested in a clade 2.3.4.4b HP H5 specific RT-qPCR [19] and additionally in a N8 specific RT-qPCR to confirm the H5N8 HPAI subtype.

Full-genome sequencing of AIV-positive samples was executed as previously described [20] via a nanopore-based multiplexed amplification method with the Rapid Barcoding Kit (SQK-RBK004, ONT) using a R9.4.1 flow cell on a Mk1C MinION platform (Oxford Nanopore Technologies, ONT, Oxford, UK) directed according to the manufacturer’s instructions. Live basecalling of the raw data with Guppy (v.4.3.4, ONT) was followed by a demultiplexing, quality check, and trimming step to remove low quality, primer, and short (<50 bp) sequences. Full-genome consensus sequences were generated in a map-to-reference approach utilizing MiniMap2 [21]. Polishing of the final genome sequences was done in Geneious Prime (Biomatters, New Zealand). The coding sequences of the eight segments were concatenated and maximum likelihood (ML) trees thereof were generated with RAxML [22] utilizing the GTR GAMMA model with rapid bootstrapping and search for the best scoring ML tree together with 1000 bootstrap replicates. The results of ML analysis were used for time-scaled Bayesian phylogenetic interference calculation with BEAST (V1.10.4) software package [23] using a GTR GAMMA substitution model, an uncorrelated relaxed clock with a lognormal distribution and coalescent constant population tree models. Chain lengths were set to 50 million iterations and convergence was checked via Tracer (V1.7.1). Time-scaled summary maximum clade credibility trees (MCC) with 10% for the post burn-in posterior were created using TreeAnnotator (V1.10.4) and visualized with FigTree (V1.4.4) showing posterior confidence values at each branch.

To get an impression of the within-farm spread of HPAI on index farm A (i.e., in the compartment with 19-week-old hens), a simple SIR model (susceptible, infectious, removed, [24]) was implemented in R [10]. It was assumed that a single chicken was infected on day -14 (Figure 4). It was further assumed that a chicken becomes infectious two days post infection (latent period according to [11]) and that all infected chickens die after four to five days [13]. Dead chicken left the status “infectious” in the model. The infectious period was thus set to 2.5 days. The secondary attack rate per day and chicken with the status “infectious” was modeled using a Poisson distribution with an average number of events of λ = 4.4 [13]. The simulation was run with 1000 iterations, the mean of infected and dead chickens per day was calculated and rounded. Results are presented in Table 2 and Figure 4, respectively.

To assess the probability of infection of a batch of chicken at the day when it left the index farm, the number of chickens included in the batches was determined from all deliveries originating from the specific batch, be it through direct delivery to a single customer, during sales tours or after relocation to the branch farm with subsequent distribution. The number of infected birds on the respective day was derived from the model describing the spread of disease within the affected compartment with 19-week-old chicken on farm A (Table 2). The probability of a batch to be infected and thus at least one secondary outbreak was calculated as the probability of at least one infected bird to be present in a batch according to the following equation

P = 1 − ((1 − (n_inf_/N))^n^ (1)

with n_inf_ denoting the number of infected chickens in the farm compartment, N the population size, and n the size of the batch.

## Figures and Tables

**Table 1:** Contacts of farm A and branch farm A_1_ and resulting secondary outbreaks.

Federal State	No. of Contacts	No. of Outbreaks
Baden-Württemberg	113 ^1^	64 ^1^
Bavaria	7 ^2^	1 ^2^
Hesse	1	0
Thuringia	187	35
Saxony	2	2
North Rhine-Westphalia	4 ^3^	3 ^3^
Sum	314	105

^1^ six additional contacts and outbreaks due to further trade of contact farm. ^2^ one additional contact and outbreak revealed by molecular analysis (Figure 3, “F”). ^3^ one additional contact and outbreak probably due to close vicinity of the affected farm with a direct contact holding (trade) of outbreak farm A (Figure 1, “1.”).

**Table 2:** Infection probability of batches of chickens leaving farm A.

Day ^1^	Population Size	Infected Chickens	Batch Size	Probability ^2^
-14	13,290	1	150	0.01122
-12	13,140	1	311	0.02339
-10	12,829	10	400	0.26800
-9	12,429	14	270	0.26236
-8	12,159	37	52	0.14656
-5	12,107	295	732	1
-4	11,375	643	136	0.99963
-3	11,239	1236	25	0.94567
-2	11,214	2501	281	1
-1	10,933	5274	386	1

^1^ before the outbreak was suspected on farm A. ^2^ of the batch to be infected (i.e., at least one chicken infected).

## Data Availability

The data presented in this study are available in this article.
